# (2*E*)-2-(4-Bromo­benzyl­idene)-2,3-di­hydro-1*H*-inden-1-one

**DOI:** 10.1107/S1600536812006654

**Published:** 2012-02-17

**Authors:** Abdullah M. Asiri, Hassan M. Faidallah, Khulud F. Al-Nemari, Seik Weng Ng, Edward R. T. Tiekink

**Affiliations:** a Chemistry Department, Faculty of Science, King Abdulaziz University, PO Box 80203, Jeddah, Saudi Arabia; b The Center of Excellence for Advanced Materials Research, King Abdulaziz University, Jeddah, PO Box 80203, Saudi Arabia; c Department of Chemistry, University of Malaya, 50603 Kuala Lumpur, Malaysia

## Abstract

The title indan-1-one derivative, C_16_H_11_BrO, is planar, the r.m.s. deviation for all 18 non-H atoms being 0.071 Å. The configuration about the C=C bond [1.337 (5) Å] is *E*. In the crystal, supra­molecular layers in the *ab* plane are formed by C—H⋯O inter­actions, involving the bifurcated carbonyl O atom, as well as C—H⋯π inter­actions. The studied crystal was an inversion twin.

## Related literature

For the activity of related species for the treatment of Chagas disease, see: Vera-DiVaio *et al.* (2009 ▶).
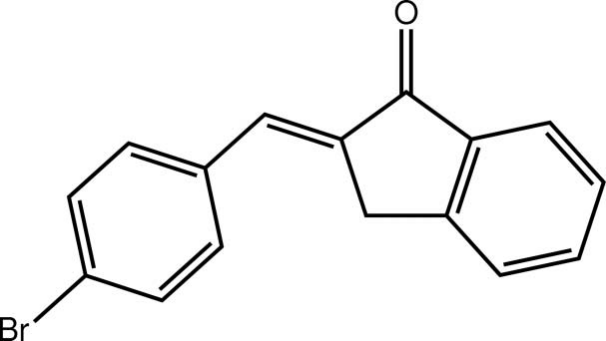


## Experimental

### 

#### Crystal data

C_16_H_11_BrO
*M* *_r_* = 299.16
Monoclinic, 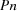

*a* = 6.1359 (5) Å
*b* = 4.7512 (4) Å
*c* = 21.310 (3) Å
β = 96.195 (9)°
*V* = 617.61 (11) Å^3^
*Z* = 2
Mo *K*α radiation
μ = 3.31 mm^−1^
*T* = 100 K
0.20 × 0.10 × 0.05 mm

#### Data collection

Agilent SuperNova Dual diffractometer with an Atlas detector
Absorption correction: multi-scan (*CrysAlis PRO*; Agilent, 2011 ▶) *T*_min_ = 0.798, *T*_max_ = 1.000
5083 measured reflections
2705 independent reflections
2455 reflections with *I* > 2σ(*I*)
*R*_int_ = 0.039

#### Refinement

*R*[*F* ^2^ > 2σ(*F* ^2^)] = 0.036
*wR*(*F* ^2^) = 0.068
*S* = 0.99
2705 reflections
164 parameters
2 restraints
H-atom parameters constrained
Δρ_max_ = 0.70 e Å^−3^
Δρ_min_ = −0.35 e Å^−3^
Absolute structure: Flack (1983 ▶), 1288 Friedel pairs
Flack parameter: 0.343 (10)

### 

Data collection: *CrysAlis PRO* (Agilent, 2011 ▶); cell refinement: *CrysAlis PRO*; data reduction: *CrysAlis PRO*; program(s) used to solve structure: *SHELXS97* (Sheldrick, 2008 ▶); program(s) used to refine structure: *SHELXL97* (Sheldrick, 2008 ▶); molecular graphics: *ORTEP-3* (Farrugia, 1997 ▶) and *DIAMOND* (Brandenburg, 2006 ▶); software used to prepare material for publication: *publCIF* (Westrip, 2010 ▶).

## Supplementary Material

Crystal structure: contains datablock(s) global, I. DOI: 10.1107/S1600536812006654/bt5818sup1.cif

Structure factors: contains datablock(s) I. DOI: 10.1107/S1600536812006654/bt5818Isup2.hkl

Supplementary material file. DOI: 10.1107/S1600536812006654/bt5818Isup3.cml

Additional supplementary materials:  crystallographic information; 3D view; checkCIF report

## Figures and Tables

**Table 1:** Hydrogen-bond geometry (Å, °) *Cg*1 is the centroid of the C2–C7 ring.

*D*—H⋯*A*	*D*—H	H⋯*A*	*D*⋯*A*	*D*—H⋯*A*
C1—H1b⋯O1^i^	0.99	2.35	3.220 (5)	146
C15—H15⋯O1^ii^	0.95	2.54	3.171 (5)	124
C1—H1*A*⋯*Cg*1^iii^	0.99	2.61	3.479 (4)	147

Symmetry codes: (i) 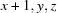; (ii) 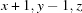; (iii) 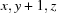.

**Fig. 1.**
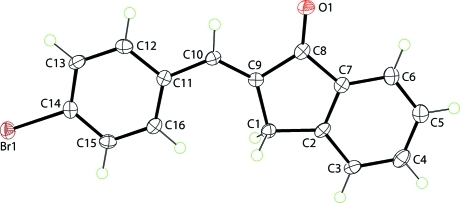
The molecular structure of (I) showing the atom-labelling scheme and displacement ellipsoids at the 50% probability level.

**Fig. 2.**
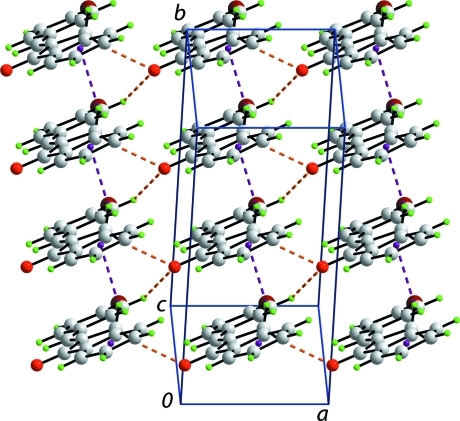
A view of the supramolecular layer in (I). The C—H···O and C—H···π interactions are shown as orange and purple dashed lines, respectively.

**Fig. 3.**
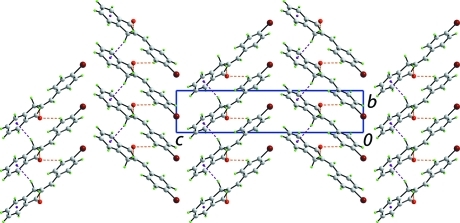
A view in projection down the a axis of the unit-cell contents of (I). The C—H···O and C—H···π interactions are shown as orange and purple dashed lines, respectively.

## supplementary crystallographic information

### Comment

The motivation for the investigation of the title compound, 2-(4-bromobenzylidene)indan-1-one (I), is its relationship to some active compounds developed for the treatment of Chagas disease (Vera-DiVaio *et al.*, 2009).

The molecule of (I), Fig. 1, is planar with a r.m.s. deviation for all 18 non-hydrogen atoms = 0.071 Å. The maximum deviations are found for the Br1 [-0.195 (1) Å] and C16 [-0.096 (3) Å] atoms. The configuration about the C9═C10 bond [1.337 (5) Å] is *E*.

In the crystal packing, C—H···O interactions involving the bifurcated carbonyl-O atom as well as C—H···π interactions link molecules into layers in the *ab* plane, Fig. 2 and Table 1. The layers stack along the *c* axis with no specific interactions between them, Fig. 2.

### Experimental

A solution of the 4-bromobenzaldehyde (1.8 g, 0.01 mol) in ethanol (20 ml) was added to a stirred solution of 1-indanone (1.3 g, 0.01 mol) in ethanolic KOH (20%, 20 ml), and stirring was maintained at room temperature for 6 h. The reaction mixture was then poured onto water (200 ml) and set aside overnight. The precipitated solid product was collected by filtration, washed with water, dried and recrystallized from its ethanol solution as blocks; *M*.pt: 453–455 K.

### Refinement

H-atoms were placed in calculated positions [C—H = 0.95 to 0.99 Å, *U*_iso_(H) = 1.2*U*_eq_(C)] and were included in the refinement in the riding model approximation. The structure was refined as a racemic twin; the Flack parameter (Flack, 1983) was explicitly refined to 0.343 (10) indicating the fractional contribution of the minor twin component. Owing to poor agreement, the (3 1 6) and (3 0 5) reflections were omitted from the final cycles of refinement.

### Crystal data

**Table:**

C_16_H_11_BrO	*F*(000) = 300
*M**_r_* = 299.16	*D*_x_ = 1.609 Mg m^−^^3^
Monoclinic, *P**n*	Mo *K*α radiation, λ = 0.71073 Å
Hall symbol: P -2yac	Cell parameters from 2365 reflections
*a* = 6.1359 (5) Å	θ = 2.9–27.5°
*b* = 4.7512 (4) Å	µ = 3.31 mm^−^^1^
*c* = 21.310 (3) Å	*T* = 100 K
β = 96.195 (9)°	Prism, orange
*V* = 617.61 (11) Å^3^	0.20 × 0.10 × 0.05 mm
*Z* = 2	

### Data collection

**Table:**

Agilent SuperNova Dual diffractometer with an Atlas detector	2705 independent reflections
Radiation source: SuperNova (Mo) X-ray Source	2455 reflections with *I* > 2σ(*I*)
Mirror monochromator	*R*_int_ = 0.039
Detector resolution: 10.4041 pixels mm^-1^	θ_max_ = 27.6°, θ_min_ = 3.4°
ω scan	*h* = −7→7
Absorption correction: multi-scan (*CrysAlis PRO*; Agilent, 2011)	*k* = −6→6
*T*_min_ = 0.798, *T*_max_ = 1.000	*l* = −27→27
5083 measured reflections	

### Refinement

**Table:**

Refinement on *F*^2^	Secondary atom site location: difference Fourier map
Least-squares matrix: full	Hydrogen site location: inferred from neighbouring sites
*R*[*F*^2^ > 2σ(*F*^2^)] = 0.036	H-atom parameters constrained
*wR*(*F*^2^) = 0.068	*w* = 1/[σ^2^(*F*_o_^2^) + (0.0265*P*)^2^] where *P* = (*F*_o_^2^ + 2*F*_c_^2^)/3
*S* = 0.99	(Δ/σ)_max_ < 0.001
2705 reflections	Δρ_max_ = 0.70 e Å^−^^3^
164 parameters	Δρ_min_ = −0.35 e Å^−^^3^
2 restraints	Absolute structure: Flack (1983), 1288 Friedel pairs
Primary atom site location: structure-invariant direct methods	Flack parameter: 0.343 (10)

### Special details

**Table:**

Geometry. All s.u.'s (except the s.u. in the dihedral angle between two l.s. planes) are estimated using the full covariance matrix. The cell s.u.'s are taken into account individually in the estimation of s.u.'s in distances, angles and torsion angles; correlations between s.u.'s in cell parameters are only used when they are defined by crystal symmetry. An approximate (isotropic) treatment of cell s.u.'s is used for estimating s.u.'s involving l.s. planes.
Refinement. Refinement of *F*^2^ against ALL reflections. The weighted *R*-factor *wR* and goodness of fit *S* are based on *F*^2^, conventional *R*-factors *R* are based on *F*, with *F* set to zero for negative *F*^2^. The threshold expression of *F*^2^ > σ(*F*^2^) is used only for calculating *R*-factors(gt) *etc*. and is not relevant to the choice of reflections for refinement. *R*-factors based on *F*^2^ are statistically about twice as large as those based on *F*, and *R*- factors based on ALL data will be even larger.

### Fractional atomic coordinates and isotropic or equivalent isotropic displacement parameters (Å^2^)

**Table:**

	*x*	*y*	*z*	*U*_iso_*/*U*_eq_	
Br1	0.50001 (3)	0.38435 (6)	0.489989 (19)	0.02212 (10)	
O1	−0.1668 (4)	1.6578 (5)	0.71975 (12)	0.0216 (6)	
C1	0.3820 (7)	1.3644 (7)	0.74899 (19)	0.0166 (9)	
H1A	0.4144	1.1744	0.7667	0.020*	
H1B	0.4993	1.4196	0.7230	0.020*	
C2	0.3597 (7)	1.5761 (7)	0.80064 (19)	0.0161 (9)	
C3	0.5184 (7)	1.6540 (7)	0.84972 (18)	0.0194 (8)	
H3	0.6596	1.5695	0.8541	0.023*	
C4	0.4630 (7)	1.8602 (7)	0.89226 (18)	0.0235 (9)	
H4	0.5677	1.9145	0.9262	0.028*	
C5	0.2575 (7)	1.9869 (8)	0.88561 (19)	0.0219 (9)	
H5	0.2247	2.1283	0.9147	0.026*	
C6	0.0998 (7)	1.9098 (8)	0.83714 (17)	0.0198 (8)	
H6	−0.0412	1.9947	0.8328	0.024*	
C7	0.1545 (6)	1.7034 (7)	0.79481 (17)	0.0166 (8)	
C8	0.0200 (7)	1.5875 (8)	0.73932 (19)	0.0162 (9)	
C9	0.1582 (6)	1.3722 (7)	0.71053 (19)	0.0137 (8)	
C10	0.0759 (6)	1.2291 (7)	0.65939 (17)	0.0155 (8)	
H10	−0.0727	1.2694	0.6449	0.019*	
C11	0.1822 (6)	1.0181 (7)	0.62235 (17)	0.0147 (8)	
C12	0.0596 (6)	0.9077 (7)	0.56840 (17)	0.0184 (8)	
H12	−0.0877	0.9670	0.5581	0.022*	
C13	0.1497 (6)	0.7144 (8)	0.53015 (17)	0.0197 (9)	
H13	0.0652	0.6404	0.4939	0.024*	
C14	0.3645 (6)	0.6305 (7)	0.54539 (17)	0.0168 (8)	
C15	0.4890 (6)	0.7281 (8)	0.59864 (18)	0.0184 (8)	
H15	0.6355	0.6652	0.6088	0.022*	
C16	0.3964 (6)	0.9197 (7)	0.63707 (17)	0.0177 (8)	
H16	0.4803	0.9856	0.6742	0.021*	

### Atomic displacement parameters (Å^2^)

**Table:**

	*U*^11^	*U*^22^	*U*^33^	*U*^12^	*U*^13^	*U*^23^
Br1	0.02551 (18)	0.02170 (16)	0.01955 (16)	0.0011 (2)	0.00433 (13)	−0.0025 (2)
O1	0.0155 (14)	0.0269 (14)	0.0218 (14)	0.0010 (11)	−0.0003 (12)	−0.0013 (11)
C1	0.020 (2)	0.017 (2)	0.0131 (19)	0.0002 (16)	0.0018 (18)	0.0031 (15)
C2	0.022 (2)	0.0155 (18)	0.0107 (19)	−0.0047 (16)	−0.0006 (18)	0.0059 (16)
C3	0.0197 (19)	0.0189 (18)	0.0184 (19)	−0.0011 (16)	−0.0036 (16)	0.0032 (16)
C4	0.032 (2)	0.022 (2)	0.0157 (19)	−0.0059 (17)	−0.0009 (17)	0.0035 (16)
C5	0.029 (2)	0.0162 (19)	0.021 (2)	−0.0011 (17)	0.0057 (18)	0.0020 (17)
C6	0.022 (2)	0.021 (2)	0.018 (2)	−0.0028 (17)	0.0079 (16)	0.0013 (16)
C7	0.020 (2)	0.0139 (17)	0.0160 (18)	−0.0033 (15)	0.0024 (16)	0.0027 (15)
C8	0.018 (2)	0.018 (2)	0.0130 (19)	−0.0058 (17)	0.0037 (18)	0.0039 (16)
C9	0.0134 (18)	0.0120 (19)	0.016 (2)	−0.0036 (14)	0.0034 (17)	0.0046 (15)
C10	0.0146 (18)	0.0200 (19)	0.0122 (17)	−0.0016 (15)	0.0025 (15)	0.0032 (16)
C11	0.019 (2)	0.0118 (17)	0.0138 (19)	−0.0043 (15)	0.0035 (16)	0.0034 (15)
C12	0.0180 (19)	0.0155 (18)	0.0215 (19)	−0.0006 (15)	0.0010 (16)	0.0050 (16)
C13	0.020 (2)	0.0188 (19)	0.0187 (19)	−0.0022 (15)	−0.0054 (17)	0.0021 (15)
C14	0.023 (2)	0.0132 (17)	0.0157 (18)	−0.0038 (15)	0.0068 (16)	0.0025 (14)
C15	0.0158 (19)	0.0207 (18)	0.0187 (18)	0.0008 (15)	0.0022 (16)	0.0051 (16)
C16	0.020 (2)	0.0183 (19)	0.0149 (18)	−0.0040 (16)	0.0012 (15)	0.0013 (16)

### Geometric parameters (Å, º)

**Table:**

Br1—C14	1.914 (4)	C7—C8	1.474 (5)
O1—C8	1.223 (5)	C8—C9	1.501 (5)
C1—C2	1.508 (5)	C9—C10	1.337 (5)
C1—C9	1.522 (5)	C10—C11	1.471 (5)
C1—H1A	0.9900	C10—H10	0.9500
C1—H1B	0.9900	C11—C16	1.398 (5)
C2—C7	1.390 (5)	C11—C12	1.406 (5)
C2—C3	1.400 (5)	C12—C13	1.382 (5)
C3—C4	1.401 (5)	C12—H12	0.9500
C3—H3	0.9500	C13—C14	1.382 (5)
C4—C5	1.391 (6)	C13—H13	0.9500
C4—H4	0.9500	C14—C15	1.378 (5)
C5—C6	1.386 (5)	C15—C16	1.387 (5)
C5—H5	0.9500	C15—H15	0.9500
C6—C7	1.398 (5)	C16—H16	0.9500
C6—H6	0.9500		
			
C2—C1—C9	103.2 (3)	C7—C8—C9	106.9 (3)
C2—C1—H1A	111.1	C10—C9—C8	119.9 (4)
C9—C1—H1A	111.1	C10—C9—C1	131.8 (4)
C2—C1—H1B	111.1	C8—C9—C1	108.3 (3)
C9—C1—H1B	111.1	C9—C10—C11	129.3 (3)
H1A—C1—H1B	109.1	C9—C10—H10	115.3
C7—C2—C3	120.1 (4)	C11—C10—H10	115.3
C7—C2—C1	112.2 (3)	C16—C11—C12	117.8 (3)
C3—C2—C1	127.7 (4)	C16—C11—C10	124.6 (3)
C2—C3—C4	118.1 (4)	C12—C11—C10	117.6 (3)
C2—C3—H3	121.0	C13—C12—C11	121.2 (4)
C4—C3—H3	121.0	C13—C12—H12	119.4
C5—C4—C3	121.2 (4)	C11—C12—H12	119.4
C5—C4—H4	119.4	C14—C13—C12	119.0 (3)
C3—C4—H4	119.4	C14—C13—H13	120.5
C6—C5—C4	120.9 (4)	C12—C13—H13	120.5
C6—C5—H5	119.5	C15—C14—C13	121.8 (3)
C4—C5—H5	119.5	C15—C14—Br1	118.3 (3)
C5—C6—C7	118.0 (4)	C13—C14—Br1	119.9 (3)
C5—C6—H6	121.0	C14—C15—C16	118.8 (3)
C7—C6—H6	121.0	C14—C15—H15	120.6
C2—C7—C6	121.7 (3)	C16—C15—H15	120.6
C2—C7—C8	109.4 (3)	C15—C16—C11	121.5 (3)
C6—C7—C8	128.8 (4)	C15—C16—H16	119.3
O1—C8—C7	126.5 (4)	C11—C16—H16	119.3
O1—C8—C9	126.6 (4)		
			
C9—C1—C2—C7	−1.2 (4)	O1—C8—C9—C1	177.3 (4)
C9—C1—C2—C3	−179.9 (4)	C7—C8—C9—C1	−1.2 (4)
C7—C2—C3—C4	0.4 (5)	C2—C1—C9—C10	−178.9 (4)
C1—C2—C3—C4	179.0 (4)	C2—C1—C9—C8	1.5 (4)
C2—C3—C4—C5	−0.8 (5)	C8—C9—C10—C11	177.7 (4)
C3—C4—C5—C6	1.0 (6)	C1—C9—C10—C11	−1.9 (7)
C4—C5—C6—C7	−0.7 (6)	C9—C10—C11—C16	2.8 (6)
C3—C2—C7—C6	−0.2 (6)	C9—C10—C11—C12	−177.2 (4)
C1—C2—C7—C6	−178.9 (3)	C16—C11—C12—C13	−1.8 (5)
C3—C2—C7—C8	179.3 (3)	C10—C11—C12—C13	178.2 (3)
C1—C2—C7—C8	0.5 (5)	C11—C12—C13—C14	−0.3 (5)
C5—C6—C7—C2	0.3 (5)	C12—C13—C14—C15	1.9 (5)
C5—C6—C7—C8	−179.0 (4)	C12—C13—C14—Br1	−175.5 (3)
C2—C7—C8—O1	−178.1 (4)	C13—C14—C15—C16	−1.3 (5)
C6—C7—C8—O1	1.3 (7)	Br1—C14—C15—C16	176.2 (3)
C2—C7—C8—C9	0.4 (4)	C14—C15—C16—C11	−1.0 (5)
C6—C7—C8—C9	179.9 (4)	C12—C11—C16—C15	2.5 (5)
O1—C8—C9—C10	−2.3 (6)	C10—C11—C16—C15	−177.5 (3)
C7—C8—C9—C10	179.1 (3)		

### Hydrogen-bond geometry (Å, º)

Cg1 is the centroid of the C2–C7 ring.

**Table:**

*D*—H···*A*	*D*—H	H···*A*	*D*···*A*	*D*—H···*A*
C1—H1b···O1^i^	0.99	2.35	3.220 (5)	146
C15—H15···O1^ii^	0.95	2.54	3.171 (5)	124
C1—H1*A*···*Cg*1^iii^	0.99	2.61	3.479 (4)	147

Symmetry codes: (i) *x*+1, *y*, *z*; (ii) *x*+1, *y*−1, *z*; (iii) *x*, *y*+1, *z*.
